# Revolving images and multi-image keys open new horizons in descriptive taxonomy: ZooKeys working examples

**DOI:** 10.3897/zookeys.328.6171

**Published:** 2013-09-03

**Authors:** Pavel Stoev, Lyubomir Penev, Nesrine Akkari, David Koon-Bong Cheung, Henrik Enghoff, Adam Brunke, Carina Mara de Souza, Thomas Pape, Daniel Mietchen, Terry Erwin

**Affiliations:** 1 National Museum of Natural History, Bulgarian Academy of Sciences and Pensoft Publishers, 12, Prof. Georgi Zlatarski St., 1700 Sofia, Bulgaria; 2 Institute of Biodiversity and Ecosystem Research, Bulgarian Academy of Sciences and Pensoft Publishers, 12, Prof. Georgi Zlatarski St., 1700 Sofia, Bulgaria; 3 Natural History Museum of Denmark (Zoological Museum), University of Copenhagen, Universitetsparken 15, Copenhagen, Denmark, 2100; 4 Department of Animal Biology, Institute of Biology, State University of Campinas (UNICAMP), Barão Geraldo, Campinas, São Paulo, Brazil, P.O.B. 6109, 13083-970; 5 Museum für Naturkunde – Leibniz-Institut für Evolutions- und Biodiversitätsforschung, Invalidenstraße 43, 10115 Berlin, Germany and Pensoft Publishers, 12, Prof. Georgi Zlatarski St., 1700 Sofia, Bulgaria; 6 Department of Entomology, MRC 187, National Museum of Natural History, Smithsonian Institution, P. O. Box 37012, Washington, DC 20013-7012 USA

Illustrations are indispensable in the recognition of species, irrespective of whether they are used for taxonomic, biological or conservational purposes. With the development of Web 2.0 and Open Access publishing, the demand for image quality and methods for visualizing taxonomic traits has significantly increased. Since its launch in 2008, ZooKeys has been supporting the development of new methods in taxonomy and advocating new tools for the visualization of taxonomic content. Publishing interactive keys as part of taxonomic revisions has become a routine practice for the journal (see e.g., Sharkey et al. 2009a, b, van Noort and Johnson 2009, Stoev et al. 2010, Cerretti et al. 2012). With the development of the journal’s web platform, ZooKeys has also started to support the publication of various types of multimedia and audio records, either as supplementary materials or as files embedded in the paper itself (see e.g. Hertz et al. 2012, Faulwetter et al. 2013, Akkari et al. 2013).

A novel approach for the visualization of taxonomic traits exemplified by a modern revision of millipedes of the genus *Ommatoiulus* is published in this issue of ZooKeys (Akkari et al. 2013), along with a detailed technical description and applied workflow (Cheung et al. 2013). It presents an innovative case study aiming to overcome the challenges faced by taxonomists in describing the complex structures essential for species description and identification. The authors use multiple techniques, including an interactive key and a new rotatable scanning electron microscope (rSEM) model to meet these challenges. They present a key design which prioritizes the visual delivery of taxonomic information via interactive media, including line drawings, photographs and scanning electron micrographs of the male genitalia (gonopods). The development of rSEM is widely accessible, requiring no more than access to a scanning electron microscope and some form of software for image integration (Flash, Java Script based programs, etc.). This technique is used for the first time to enhance taxonomic descriptions and allows the structure in question to be seen from multiple angles of view.

The yet slow rate of utilization and acceleration of multimedia in taxonomic research is very likely due to the perception that sophisticated imaging requires special software, e-infrastructure, and significant funding. The method applied here proves that wrong as it enables the visualization of important taxonomic characters in great detail from various angles and can be achieved comparatively effortlessly with conventional technology and software. In addition to providing new insights on the application of SEM and bringing a touch of modernism to taxonomic studies in general, the use of detailed rotating illustrations for small and complex anatomical structures, such as millipede genitalia, revealed diagnostic characters that would have remained unnoticed with conventional methods. The use of these rSEM as a replacement for static illustrations in taxonomic revisions puts us one step closer to the development of a software capable of automatically extracting morphological character data from images of organisms and providing users with the species name (La Salle et al. 2009). Though conceived only to better visualize surface structures, the rSEM model is in a way proving that creating three-dimensional imaging libraries and virtual specimen collections is possible, with a rapid access to “cybertypes,” a term recently introduced by Faulwetter et al. (2013).
